# The Impact of Pectin Supplementation on Intestinal Barrier Function in Healthy Young Adults and Healthy Elderly

**DOI:** 10.3390/nu11071554

**Published:** 2019-07-09

**Authors:** Ellen Wilms, Daisy M.A.E. Jonkers, Huub F.J. Savelkoul, Montserrat Elizalde, Lea Tischmann, Paul de Vos, Ad A.M. Masclee, Freddy J. Troost

**Affiliations:** 1 Division Gastroenterology-Hepatology, Department of Internal Medicine, NUTRIM School of Nutrition and Translational Research in Metabolism, Maastricht University Medical Center+, 6202 AZ Maastricht, The Netherlands; 2 Top Institute Food and Nutrition, 6700 AN Wageningen, The Netherlands; 3 Cell Biology and Immunology Group, Department of Animal Sciences, Wageningen University, 6708 WD Wageningen, The Netherlands; 4 Department of Nutrition and Movement Sciences, NUTRIM School of Nutrition and Translational Research in Metabolism, Maastricht University Medical Center+, 6200 MD Maastricht, The Netherlands; 5 Department of Pathology and Medical Biology, Section Immunoendocrinology, University of Groningen, University Medical Center Groningen, 9713 GZ Groningen, The Netherlands; 6 Department of Food Innovation and Health, Centre for Healthy Eating and Food Innovation, Maastricht University, 5911 AA Venlo, The Netherlands

**Keywords:** aging, dietary fiber, intestinal permeability, tight junctions, defense, gastrointestinal, tolerance

## Abstract

Intestinal barrier function is suggested to decrease with aging and may be improved by pectin intake. The aim of this study was to investigate the effects of four weeks pectin supplementation on gastrointestinal barrier function in vivo and ex vivo in different age groups. In a randomized, double-blind, placebo-controlled, parallel study, 52 healthy young adults (18–40 years) and 48 healthy elderly (65–75 years) received 15 g/day pectin or placebo for four weeks. Pre- and post-intervention, in vivo gastrointestinal permeability by a multisugar test, and defense capacity in mucosal samples were assessed. Sigmoid biopsies were collected post-intervention from subgroups for Ussing chamber experiments and gene transcription of barrier-related genes. Pectin intervention did not affect in vivo gastroduodenal, small intestinal, colonic, and whole gut permeability in young adults nor in elderly (*p* ≥ 0.130). Salivary and fecal sIgA and serum IgA were not significantly different between pectin versus placebo in both age groups (*p* ≥ 0.128). In both young adults and elderly, no differences in transepithelial electrical resistance and fluorescein flux (*p* ≥ 0.164) and relative expression of genes analyzed (*p* ≥ 0.222) were found between pectin versus placebo. In conclusion, intestinal barrier function was not affected by four weeks pectin supplementation neither in healthy young adults nor in healthy elderly.

## 1. Introduction

An intact epithelial barrier is important for intestinal health and general well-being [1,2]. Epithelial cells are sealed by a junctional complex, which permits selective entry of nutrients, ions, and water while restricting permeation of bacteria and their products. An increased permeability can lead to translation of luminal antigens and thereby to intestinal and systemic inflammation. Consequently, intestinal barrier dysfunction has been associated with a variety of intestinal and systemic diseases [2] and with aging [3,4,5]. Interest in nutritional interventions to improve intestinal barrier function is increasing. Functional foods, which can be applied in targeted nutrition strategies, are added foods or ingredients that may provide health benefits beyond basic nutritional impact and/or reduce the risk of disease [6]. Examples of functional foods are food items enriched with dietary fibers. Pectin is a complex polysaccharide originating from cell walls of, for example, citrus peel, apple, and sugar beet pulp [7,8] and is composed of galacturonic acid, of which the residues are substituted with methyl esters at the C6-carboxyl group and rhamnogalacturonan [9]. In addition, sugar beet pectin as compared to, for example, citrus and apple pectins, comprises acetylation of homogalacturonan. In the upper gastrointestinal (GI) tract, pectin is resistant to digestion and hydrolysis. Because of the complex structure, pectin serves as substrate for fermentation by the microbiota in both the proximal and distal colon, resulting in the production of beneficial short-chain fatty acids (SCFAs) [8,10,11]. Pectin may impact the intestinal epithelial barrier indirectly, by modulating the colonic microbial composition and activity, and/or directly act on the epithelial cells [12,13]. Especially, the SCFA butyrate has been shown to both protect and repair intestinal barrier function, possibly via beneficial effects on junctional proteins and underlying signaling cascades [14]. Moreover, dietary fibers are suggested to reinforce intestinal barrier function through modulating the enteric immune system. It has been shown that prebiotics can be sensed by dendritic cells and in some cases selectively be transferred to the lamina propria via specialized epithelial cells (i.e., microfold (M) cells), thus signaling to the gut-associated lymphoid tissue [15]. Pectin-enhanced diets have been shown to improve intestinal barrier function, as reflected by decreased small intestinal permeability in infants with persistent diarrhea [16] and rat studies [17,18] compared with control diets, whereas data on colonic permeability and responses to a potential stressor are not available. Furthermore, studies on the effects of pectin on mucosal defense capacity in healthy adults and elderly are lacking. 

Within the development of functional foods to target specific health concerns, it is important to study the impact of nutrition in relevant subgroup(s) [19]. For this reason, we included two different age groups: young adults and elderly. The purpose of this study was to investigate both the functional and structural effects of pectin on GI barrier function in vivo and ex vivo in young adults and elderly. The primary aim was to investigate the effects of four weeks pectin supplementation on segment-specific intestinal permeability in vivo, stratified for age group. Secondly, we aimed to investigate the effects of four weeks pectin supplementation on ex vivo stressed and unstressed intestinal barrier function, the expression of intestinal barrier related genes and mucosal defense parameters, all stratified for age group. We hypothesized that four weeks pectin supplementation improves intestinal barrier function and mucosal defense capacity in healthy subjects, while we expect effects to be most pronounced in the elderly. 

## 2. Materials and Methods 

The Medical Ethics Committee of the Maastricht University Medical Center+ approved this study, which has been designed and performed in accordance with the Declaration of Helsinki (latest amendment of 2013, Fortaleza, Brazil) and Dutch Regulations on Medical Research involving Human Subjects (1998). The study was performed at the Maastricht University Medical Center+ from March 2015 until April 2016. The trial has been registered in the Clinical Trials register (NCT02376270). All participants gave written informed consent before prior to participation. 

### 2.1. Subjects

Healthy men and women with a body mass index (BMI) between 20–30 kg/m² were recruited from two age groups by advertising, including young adults between 18–40 years of age and elderly between 65–75 years of age. Key exclusion criteria included the presence of GI symptoms, history of any chronic disorder or major surgery which potentially limited participation or completion of the study, abdominal surgery interfering with GI function, self-reported human immunodeficiency virus, average alcohol consumption of >20 alcoholic units per week, smoking, pregnancy, lactation, blood donation 90 days prior to the study, use of antibiotics, antifungal medication, probiotics or prebiotics 90 days before the start of the study, history of side effects towards pro- or prebiotic supplements, and use of nonsteroidal anti-inflammatory drugs. Use of other medication or dietary supplements was reviewed by a medical doctor, who decided on in- or exclusion based on the medications or supplements used. Included subjects using medication had to use a stable dose. Moreover, serum C-reactive protein concentrations were determined to exclude inflammation and infections, and they were measured by immunoturbidimetric assay using Cobas 6000 analyzer (Roche, Mannheim, Germany).

### 2.2. Study Design

**Figure 1:**
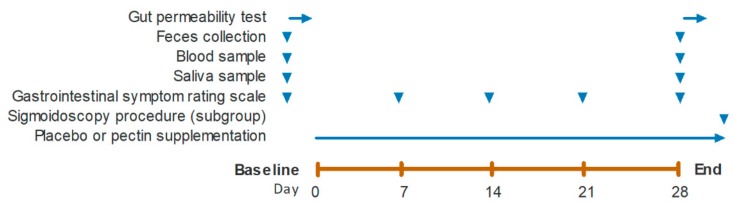
Timeline of the intervention period. Gut permeability test, feces collection, blood and saliva sampling, gastrointestinal symptom rating scale, sigmoidoscopy procedure, and placebo or pectin supplementation were completed at the days as indicated by arrows. Intake of supplements continued until all measurements were finished.

This study was designed as a randomized, double-blind, placebo-controlled, parallel-group study. Per age group, randomization was performed to assign participants to the placebo or the pectin intervention arm. An independent person generated both lists, for the young adults and the elderly, of random allocations using a computerized procedure. All study participants and investigators were blinded to intervention allocations until analyses were completed. Participants in the pectin group received 15 g/day of sugar beet derived pectin (GENU^®^ BETA pectin, CP Kelco Germany GmbH, Grossenbrode, Germany) for four weeks. Participants in the placebo group received 15 g/day of maltodextrin (GLUCIDEX^®^ IT 12, Roquette Frères, Lestrem, France) for four weeks. Fifteen grams daily were given, as this is considered a prebiotic dosage in the higher physiological range with a minimal risk of side effects [20,21]. Furthermore, four weeks is considered sufficient to strengthen the barrier function by direct effects or changes in intestinal microbiota composition and activity [22,23]. Both pectin and placebo were supplemented as dry powders free from off-flavors and odors, and they were packed in closed sachets of a single dose of 7.5 g. Subjects were asked to ingest the supplements twice daily, before breakfast in the morning and before diner in the evening, dissolved in approximately 200 mL of tap water, and mixed with flavored syrup (Karvan Cévitam^®^, Koninklijke De Ruijter B.V., Zeist, The Netherlands). Time of consumption had to be recorded, and empty and remaining sachets were returned to the investigator. At baseline and after four weeks pectin or placebo supplementation, segment-specific gut permeability tests were performed, and bio samples were collected (Figure 1). Fecal samples were collected at home, stored at −20 °C until arrival at the study site, and immediately stored at –80 °C. After fasting overnight, venous blood and saliva samples were collected and stored at –80 °C until further use. Additionally, the GI symptom rating scale (GSRS) was completed at baseline and at weekly intervals to check for GI tolerance. Due to the invasive character, a flexible sigmoidoscopy without bowel preparation was performed only at the end of each intervention and in subgroups of the young adults and elderly. A standard flexible colonoscope was inserted, and 12 biopsy specimens were taken from the sigmoid colon region with a jumbo biopsy forceps (Boston Scientific, Kerkrade, The Netherlands). Seven samples were kept viable in pre-oxygenated Krebs–Ringer bicarbonate (KRB) solution on melting ice and directly transported to the laboratory for Ussing chamber experiments. Five tissue samples were snap-frozen in liquid nitrogen and stored at −80 °C for later analyses. 

### 2.3. Gut Permeability Test 

Segment-specific permeability of the GI tract was assessed by a multisugar test as validated by van Wijck et al. [24,25]. One day prior to testing, as well as during the test, subjects were instructed to refrain from excessive physical exercise and alcohol consumption. After fasting overnight, a mix of water-soluble, nondegradable sugar probes were ingested, comprising 1 g sucrose (Van Gilse, Dinteloord, The Netherlands), 1 g lactulose (Centrafarm Services, Etten-Leur, The Netherlands), 0.5 g mannitol (Roquette, Lestrem, France), 1 g sucralose (Tate and Lyle Ingredients Americas, Decatur, IL, USA), and 1 g erythritol (Now Foods, Bloomindale, IL, USA), dissolved in 200 mL tap water. After ingestion, participants collected 24 h urine output in two separate fractions: 0–5 h and 5–24 h, respectively. During the first 5 h of urine collection, participants were asked to refrain from any food or drinks, except for water ad libitum. Thereafter, participants were allowed to eat and drink as preferred, except for sucralose-containing foods. When urine was delivered to the researcher, volumes of urine fractions were determined, and urine aliquots were frozen at −80 °C until analysis. Sugar probes were analyzed by isocratic ion-exchange high-performance liquid chromatography with mass spectrometry as described previously [24,25]. Gastroduodenal permeability was determined by sucrose excretion in 0–5 h urine, whereas small intestinal permeability was measured by calculating the lactulose to mannitol (L/M) ratio in 0–5 h urine. Sucralose to erythritol (S/E) ratios in 5–24 h and 0–24 h urine were used as indicators for colonic and whole gut permeability, respectively.

### 2.4. Mucosal Defense Parameters

For total secretory immunoglobulin A (sIgA) determination, fecal samples were thawed, 1:5 diluted with sodium chloride, incubated for 96 h, and measured by radial immunodiffusion using a commercial test kit (Binding Site, Birmingham, United Kingdom). Immunoglobulin A (IgA) subclasses IgA1 and IgA2 in serum and saliva samples were quantified by enzyme-linked immunosorbent assay. To this end, high-binding 96-well plates (Greiner Bio one 655061, Monroe, NC, USA) were coated with goat anti-human IgA-antibody preparation (Southern Biotech, 2050-01, Birmingham, United Kingdom), which was diluted in phosphate-buffered saline (PBS) at a coating concentration of 1 µg/mL for serum detection and 0.1 µg/mL for saliva detection, and were incubated overnight at 4 °C. The plates were blocked with 5% fat-free milk powder in PBS at 150 µL/well for 1–2 h at room temperature (RT). After washing three times with wash buffer (PBS + 0.05% Tween-20, Merck, Darmstadt, Germany), a total of 100 µL IgA1 or IgA2 standards and test samples per well were applied on separate plates for 1 h at 37 °C. Standard curves were set up on each plate, ranging from 200 to 0.2 ng/mL for both IgA1 and IgA2. Serum samples were diluted in Universal Casein Diluent in PBS (PBSC) at 1:32,000 and 1:64,000 in IgA1 plates and at 1:400 and 1:800 in IgA2 plates. Saliva samples were diluted in PBSC at 1:5000 and 1:10,000 on IgA1 plates and at 1:2000 and 1:10,000 on IgA2 plates. After washing four times with wash buffer, secondary antibodies specific for human IgA1 (mouse anti-human IgA1 (at 1:5000 in PBSC) or mouse anti-human IgA2 (at 1:2000 in PBSC) (Southern Biotech, 9130-08 and 9140-08, respectively) were added at 100 µL/well and incubated for 1 h at RT. After washing four times with wash buffer, 100 µL/well streptavidin-horseradish peroxidase (Southern Biotech, 7100-05), diluted in PBSC at 1:5000, was added to the plates and incubated for 45 min at RT while covered with aluminum foil. After washing six times with wash buffer, 100 µL/well 3,3’,5,5’-tetramethylbenzidine (TMB) substrate solution (SDT, Baesweiler, Germany) was added to the plates and incubated for 15 min at RT while covered with aluminum foil. The reaction was stopped by adding 2% HCL solution and measured in a Filtermax microplate reader (Molecular Devices, San Jose, CA, USA) at 450 nm minus 620 nm as a reference value. After applying five-parameter logistic transformation, the data were calculated according to best fit on the standard curve.

### 2.5. Gastrointestinal Tolerance 

The GSRS was completed at weekly intervals to check for GI tolerance. This instrument contained 15 items, and each item was graded by using a seven-point Likert-type scale where 1 represents absence of troublesome symptoms and 7 represents very troublesome symptoms. The items were combined into five subscales depicting reflux, abdominal pain, indigestion, diarrhea, and constipation [26]. 

### 2.6. Using Chamber Experiment

Six tissue samples from the sigmoid colon were used for ex vivo Ussing chamber experiments as previously described by our group [27]. Three tissue samples were mildly stressed by adding 1 µg/mL of the mast cell degranulator Compound 48/80 (Sigma-Aldrich, St. Louis, MO, USA) to the serosaL compartment. Three non-exposed tissue samples served as unstressed controls. At *t* = 0, 1 mg/mL fluorescein (376 g/mol, Sigma-Aldrich, St. Louis, MO, USA) was added to the serosal compartment for determination of fluorescein flux to the luminal compartment. From all tissue samples, potential difference (PD), transepithelial electrical resistance (TEER), and fluorescein concentrations were determined at time point *t* = 0, 30, 60, 80, and 120 min. TEER and PD were used as quality criteria for viability. Only samples with a baseline TEER above 20 Ω/cm^2^, or those with baseline TEER between 15–20 Ω/cm^2^ and PD below 0.5 mV, were included for analyses. TEER and fluorescein concentrations are indicators of intestinal permeability. 

### 2.7. Gene Transcription of Relevant Proteins

Transcription of junctional complex related genes, as well as defense- and immune-related genes associated with barrier function or modulation thereof, were determined in colonic tissue samples. Nucleic acid extraction and purification, RNA isolation, reverse transcription, and quantitative real-time polymerase chain reaction (qRT-PCR) were performed as previously described [28]. Depending on the gene of interest, cDNA was diluted to final concentrations of 20, 40, or 80 ng/µL (Table S1). Expressions of target genes were normalized to glyceraldehyde-3-phosphate dehydrogenase (*GAPDH*) and 18S ribosomal RNA (*18S RNA*) as reference genes (Table S1). 

### 2.8. Immunofluorescence Staining of TJP1 and Occludin

Sigmoid biopsy sections (10 µm) were used for immunofluorescent staining of TJP1 and occludin as previously described by our group [29].

### 2.9. Statistical Analyses

The sample size calculation of the primary outcome (i.e., in vivo intestinal permeability) was based on the difference in urinary lactulose/mannitol ratio between inulin-enriched pasta and control pasta in young males as reported by Russo et al. [30]. A difference between treatments of 0.02, standard deviation of 0.022, alpha of 0.025, and power of 0.80 were assumed. Thereby, a minimum of 24 completers per intervention group in each age group were needed.

Intention to treat analyses were performed. Normality of the data was checked by histograms and was summarized accordingly using the median and interquartile range (IQR; 25–75th IQR) or means ± standard deviation for numerical variables as well as percentages for categorical variables. Independent-sample *t*-tests were performed for numerical variables and chi-square tests for categorical variables to test for differences between intervention groups (pectin versus placebo) in young adults and in elderly. 

Within each age group, differences between interventions were assessed by unstructured linear mixed model analyses with intervention group (pectin and placebo), time (baseline and end) and ‘intervention group × time’ as fixed factors, and correction for baseline values. Differences in longitudinal trends in TEER and luminal fluorescein between intervention groups were assessed by random intercept linear mixed model analyses with intervention group (pectin and placebo), time (*t* = 0, 30, 60, 90, and 120 min) and ‘intervention group × time’ as fixed factors, and correction for *t* = 0 values. All statistical analyses were performed for young adults and elderly separately using IBM SPSS Statistics for Windows (version 25.0, IBM Corporation, Armonk, NY, USA). A *p*-value ≤ 0.05 (two-sided) was considered statistically significant. GI symptoms, Ussing chamber experiments, and gene transcription *p*-values were corrected for multiple testing by the false discovery rate (FDR) of Benjamini–Hochberg.

## 3. Results

### 3.1. Study Subjects

**Figure 2:**
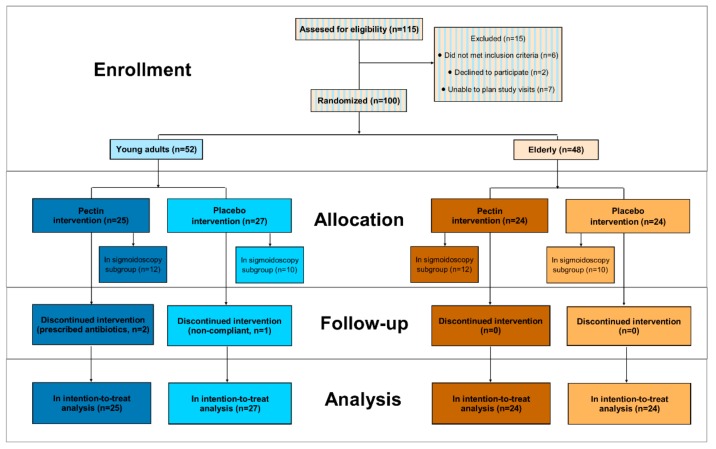
Flow diagram of the study.

After assessment of eligibility, 52 young adults and 48 elderly were enrolled in the study. Three young adults dropped out, one because of overt noncompliance and two because of antibiotic use (Figure 2). Baseline characteristics of the young adults and elderly, undergoing either pectin or placebo intervention, are shown in Table 1.

Moreover, a subgroup of 22 young adults and 22 elderly underwent a sigmoidoscopy after four weeks pectin or placebo intervention (Figure 2), of which baseline characteristics are shown in Table S2. 

### 3.2. Intestinal Permeability In Vivo

**Figure 3:**
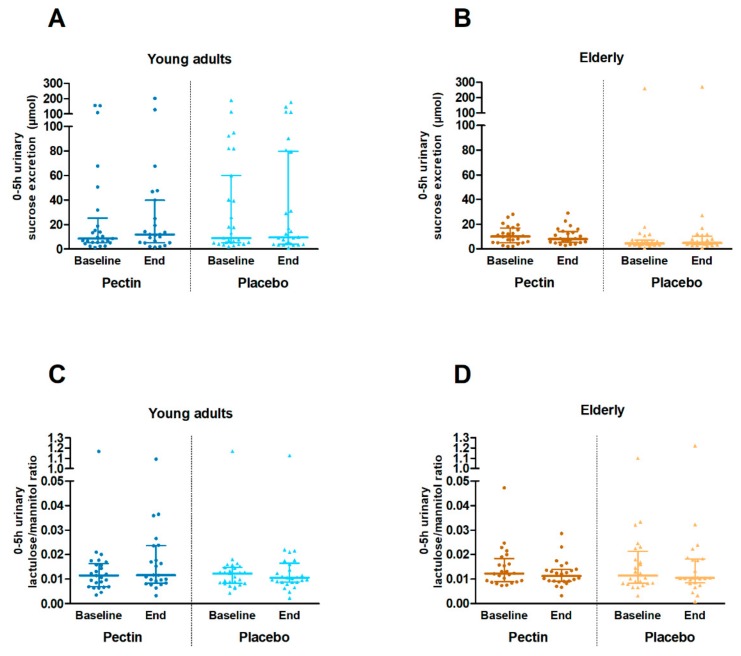
Gastroduodenal and small intestinal permeability in vivo at baseline and after four weeks of placebo (triangles) and pectin (circles) intervention in young adults and elderly. (**A**) 0–5 h urinary sucrose excretion (µmol) in young adults. (**B**) 0–5 h urinary sucrose excretion (µmol) in elderly. (**C**) 0–5 h urinary lactulose/mannitol ratio in young adults. (**D**) 0–5 h urinary lactulose/rhamnose ratio in elderly. Values are presented in scatter plots with median line and IQR (25–75th interquartile range). Sample size differences between baseline and end are due to drop-outs. Within age groups, urinary sugar excretions and ratios were compared between intervention groups with an unstructured linear mixed model and correction for baseline values.

**Figure 4:**
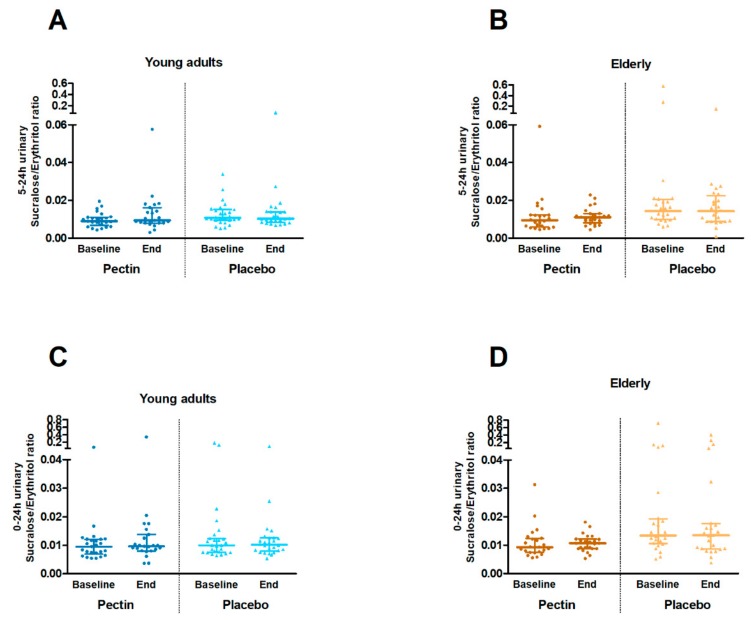
Colonic and whole gut permeability in vivo at baseline and after four weeks of placebo (triangles) and pectin (circles) intervention in young adults and elderly. (**A**) 5–24 h urinary sucralose/erythritol ratio in young adults. (**B**) 5–24 h urinary sucralose/erythritol ratio in elderly. (**C**) 0–24 h urinary sucralose/erythritol ratio in young adults. (**D**) 0–24 h urinary sucralose/erythritol ratio in elderly. Values are presented in scatter plots with median line and IQR (25–75th interquartile range). Sample size differences between baseline and end are due to drop-outs. Within age groups, urinary sugar ratios were compared between intervention groups with unstructured linear mixed models and correction for baseline values.

Gastroduodenal and small intestinal permeability, as assessed by the 0–5 h urinary sucrose excretion and 0–5 h urinary L/M ratio, respectively (Figure 3), did not differ significantly between four weeks pectin and placebo supplementation in the young adults nor in the elderly (all *p* ≥ 0.861). The 5–24 h urinary S/E ratio and 0–24 h urinary S/E ratio (Figure 4), as measures of colonic and whole gut permeability, were not significantly different between four weeks pectin vs. placebo supplementation in both young adults and elderly (all *p* ≥ 0.130). 

### 3.3. Mucosal Defense Parameters

No significant changes in salivary sIgA1 and sIgA2, serum IgA1 and IgA2, and fecal sIgA were observed between pectin or placebo intervention, neither in young adults nor in elderly (all *p* ≥ 0.128) (Figure S1). 

### 3.4. Gastrointestinal Tolerance

GI tolerance was assessed weekly by completing the GSRS questionnaire. After FDR correction for multiple testing, GI symptom scores were not significantly different between pectin and placebo supplementation in young adults nor in elderly (all *p* ≥ 0.054) (Figure S2 and Figure S3, respectively). In young adults, however, pectin intervention induced significantly higher diarrhea scores (*p* = 0.020) compared with placebo at week two only (Figure S2).

### 3.5. Intestinal Permeability Ex Vivo

**Figure 5:**
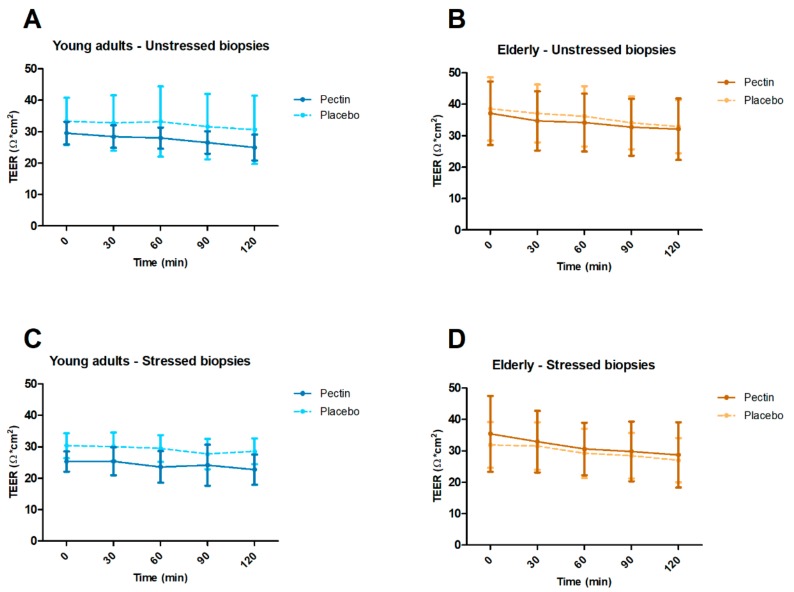
Intestinal permeability ex vivo after four weeks of pectin (fixed lines) and placebo (dashed lines) intervention in young adults and elderly. Analyses were conducted by mounting fresh sigmoid biopsies in an Ussing chamber system and assessing transepithelial electrical resistance (TEER) at *t* = 0, 30, 60, 90, and 120 min. (**A**) TEER in young adults in unstressed biopsies. (**B**) TEER in elderly in unstressed biopsies. (**C**) TEER in young adults in biopsies stressed by 1 µg/mL Compound 48/80 at *t* = 0. (**D**) TEER in elderly in biopsies stressed by 1 µg/mL Compound 48/80 at *t* = 0. Means and standard deviations are shown. Sample sizes varied because baseline values of some sigmoid biopsies did not meet quality criteria for viability. Within age groups, TEER and luminal fluorescein were compared between intervention groups with random intercept linear mixed models and correction for baseline values. *p*-values per time point were corrected for multiple testing by calculating the false discovery rate (FDR) of Benjamini–Hochberg.

**Figure 6:**
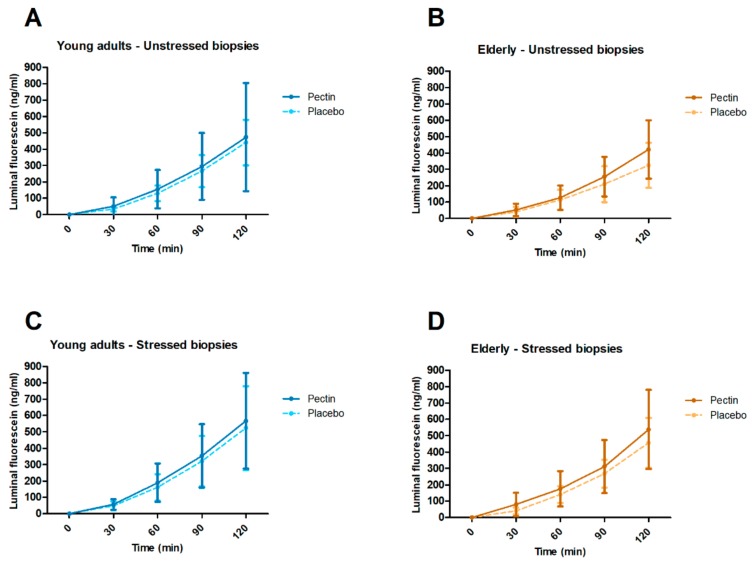
Intestinal permeability ex vivo after four weeks of pectin (fixed lines) and placebo (dashed lines) intervention in young adults and elderly. Analyses were conducted by mounting fresh sigmoid biopsies in an Ussing chamber system and assessing luminal fluorescein concentration at *t* = 0, 30, 60, 90, and 120 min. (**A**) Luminal fluorescein concentration in young adults in unstressed biopsies. (**B**) Luminal fluorescein concentration in elderly in unstressed biopsies. (**C**) Luminal fluorescein concentration in young adults in biopsies stressed by 1 µg/mL Compound 48/80 at *t* = 0. (**D**) Luminal fluorescein concentration in elderly in biopsies stressed by 1 µg/mL Compound 48/80 at *t* = 0. Means and standard deviations are shown. Sample sizes varied because baseline values of some sigmoid biopsies did not meet quality criteria for viability. Within age groups, luminal fluorescein concentrations were compared between intervention groups with random intercept linear mixed models. *p*-values per time point were corrected for multiple testing by calculating the false discovery rate (FDR) of Benjamini–Hochberg.

Using chamber experiments were done to determine ex vivo TEER and luminal fluorescein concentration as indicators of paracellular permeability in unstressed and stressed conditions. After FDR correction for multiple time points, TEER in unstressed and stressed biopsies did not significantly differ between four weeks pectin versus placebo supplementation in elderly nor in young adults (all *p* ≥ 0.226) (Figure 5). In both young adults and elderly, luminal fluorescein concentrations in unstressed and stressed biopsies did not differ significantly between four weeks pectin vs. placebo supplementation (all *p* ≥ 0.164) (Figure 6).

### 3.6. Gene Transcription of Barrier-Related Genes

Mean Cq values of both *GAPDH* and *18S RNA* did not differ between pectin and placebo intervention. *GAPDH* normalized relative expression of junctional complexes (e.g., tight junctions and adheren junctions) as well as defense- and immune-related (e.g., human defensins, cytokines, and toll-like receptor) genes in sigmoid biopsies of young adults and elderly after four weeks pectin or placebo intervention are shown in Table 2. After FDR correction for multiple testing, in both young adults and elderly, no significant differences were found between pectin vs. placebo intervention (all *p* ≥ 0.222) in the relative expression of all genes analyzed. Moreover, analyses on *18S RNA* normalized gene expressions resulted in the same conclusions.

### 3.7. Immunofluorescence Staining of TJP1 and Occludin

Visual inspection of representative immunofluorescence staining of *TJP1* (Figure S4) and occludin (Figure S5) in sigmoid biopsy sections showed no apparent differences between four weeks pectin versus placebo supplementation in young adults nor in elderly. These observations are in line with quantitative analyses of *TJP1* and occludin gene transcription levels as reported in Table 2 as well as with the functional analyses performed.

## 4. Discussion

In the current study, the impact of pectin on the functional and structural GI barrier in young adults and elderly has been investigated in vivo and ex vivo. We showed that GI segment-specific permeability, intestinal permeability ex vivo, expression of barrier-related genes, and parameters of mucosal defense were not significantly improved by four weeks pectin supplementation neither in healthy young adults nor in healthy elderly.

The present study was designed based on the previously described features of pectin intake. It may strengthen the highly dynamic epithelial barrier directly by interacting with tight junction proteins and indirectly via modulating the colonic microbial composition and activity, which is known to affect intestinal homeostasis and barrier function. An intervention period of four weeks should be adequate to both directly and indirectly modulate intestinal barrier function. Moreover, sugar beet pectin was chosen because of the complex structure, which causes it to be fermented in both the proximal and distal colon. Saccharolytic fermentation (i.e., fermentation of dietary fibers) may inhibit fermentation of proteins in the distal colon due to substrate competition, thereby lowering the production of mostly toxic compounds that result from proteolytic fermentation. The only previous human study on pectin and intestinal permeability in vivo showed that one-week supplementation with pectin (4 mg/kg body weight) improved small intestinal permeability (i.e., decreased 0–5 h urinary L/M ratio) in infants with persistent diarrhea [16]. In our study, we found no significant effects of four weeks pectin intake on small intestinal permeability, as determined by the 0–5 h urinary L/M ratio, in healthy adults and healthy elderly. Because the type and dosage of pectin, intervention duration, and target populations of both human studies differed, adequate comparison of these studies is difficult. 

We also showed that gastroduodenal, colonic, and whole gut permeability, as determined by the multisugar test, were not significantly affected by four weeks pectin supplementation. This may be due to the well-functioning intestinal barrier at baseline in both the healthy young adults and the elderly, although the intestinal epithelium is often exposed to stressors such as alcohol, high-fat diet, medication use, psychological and psychosocial stress, etc. In the current study, subjects were instructed to maintain their habitual diet. As we did not actually monitor food intake over the study period, we cannot exclude that the intake of 15 g/day pectin or placebo may have impacted food intake. Furthermore, it should be noted that the inter-individual variations were rather high, although this was in accordance with previous observations [31]. This stresses the importance of assessments within subjects, as was done in the current study, with measurements of the intestinal barrier before and after the intervention period. As other dietary fibers (i.e., galacto-oligosaccharides) have been found to improve colonic permeability in obese subjects [32], based on our results, we cannot exclude potential impact of pectin on intestinal barrier function in more susceptible (sub)groups of adults or elderly.

To further examine the effect of pectin in stressed conditions, sigmoid biopsies were collected at the end of each intervention in subgroups of the young adults and the elderly. Mucosal tissue samples were used to determine intestinal permeability ex vivo in Ussing chamber experiments. We used Compound 48/80 to induce a mild stress as reflected by an increase in luminal fluorescein concentrations. Though, tissue TEER and mucosal fluorescein permeation were not affected by the four weeks pectin versus placebo supplementation in the unstressed nor in the stressed condition. Ganda Mall et al. [33] exposed sigmoid biopsies of elderly with GI symptoms and of healthy adults with dietary fibers (i.e., yeast-derived beta-glucan and wheat-derived arabinoxylan) before adding Compound 48/80. Especially in elderly with GI symptoms, beta-glucan was found to attenuate the hyperpermeability induced by Compound 48/80 as reflected by both higher TEER and lower mucosal to serosal fluorescein concentrations. Differences with the current study may be explained by the more vulnerable elderly population (i.e., with GI symptoms) and exposure to beta-glucan in vitro rather than in vivo.

Relative expression levels of junctional complex, defense, and immune related genes in sigmoid tissue samples also showed no significant differences between the sugar beet derived pectin and placebo supplementation in any of the age groups after FDR correction. This was further supported by immunofluorescence staining of TJP1 and occludin in representative sigmoid biopsy sections. Interestingly, in rats which were selected by their response to a high-fat diet by gaining weight, subsequent high-fat diet supplemented with apple-derived pectin versus normal high-fat diet resulted in lower interleukin (*IL*)-6, tumor necrosis factor-α and *TLR*4, and higher *IL*-10 and claudin-1 mRNA levels in ileal tissue, suggestive of an anti-inflammatory activity of this pectin [18]. However, possible disturbances in intestinal barrier function induced by the high-fat diet, differences in pectin source, and no corrections for multiple testing may explain, at least in part, different effects of the rat study when compared to the current human intervention study. Furthermore, in a mice model of acute pancreatitis, low-methoxyl pectin was found to upregulate occludin, *TJP1*, and defensin beta 1 as well as downregulate tumor necrosis factor-α, *IL*-β, and *IL*-6 relative mRNA levels in ileal and colonic tissue, pointing towards restoration of acute pancreatitis-associated disruption of the intestinal barrier [34]. This is not in line with our observations, probably caused by the difference between acute pancreatitis-induced animals versus healthy human participants and variation in degrees of methylation. 

The effects of four weeks pectin supplementation on mucosal defense capacity was further studied by assessing salivary sIgA1 and sIgA2, fecal sIgA, and serum IgA1 and IgA2, demonstrating no significant effect of the pectin intervention in any of the age groups. Production of sIgA in the human intestine in absolute quantities exceeds that of all other antibody classes together [35], and IgA can be seen as a key antibody class for the first line of defense in mucous membranes. Human studies investigating the effects of other dietary fibers on human mucosal defense, as assessed by salivary and fecal sIgA in vivo, have been performed previously [36,37,38,39]. However, to our knowledge, this is the first human study investigating the effects of sugar beet pectin supplementation on IgA levels. In a rat study comparing pectin (unspecified origin) versus cellulose-supplemented diets, higher serum IgA concentrations in pectin-supplemented rats were found [40], although sIgA was not determined. Conflicting results between rat and human studies can be caused by differences in pectin source or normal physiological processing of the IgA molecule, and the fact that only humans have two isotypes of IgA that are differentially regulated and distributed. 

GI symptoms were determined throughout the four weeks pectin supplementation period to monitor GI tolerance. Although pectin, in comparison to placebo, did not alter any GI symptom score in the elderly, pectin caused an increase in the diarrhea score after two weeks pectin intake by young adults. After four weeks of pectin intervention, diarrhea decreased and was no longer significantly different compared to placebo, illustrating habituation to 15 g/day of pectin supplementation in young adults. This habituation period is in line with previous findings on dietary fibers and the occurrence of gastrointestinal symptoms [41,42], and it may, in the case of pectin supplementation, be due to increased microbial fermentation and/or increased viscosity in the colonic lumen. 

## 5. Conclusions

In conclusion, by using a combined in vivo and ex vivo approach, we consistently showed that intestinal barrier function was not affected by four weeks sugar beet pectin supplementation neither in healthy young adults nor in healthy elderly. As there are clear leads in literature that dietary fibers may improve the intestinal barrier, but clinical data are still limited, further human intervention studies are needed to explore potential effects of pectin and other dietary fibers in patients with an impaired intestinal barrier function. 

## Figures and Tables

**Table 1:** Baseline characteristics of the total sample of young adults (*n* = 52) and elderly (*n* = 48), undergoing either placebo or pectin intervention.

Parameter	Young Adults (*n* = 52)			Elderly (*n* = 48)		
	Pectin (*n* = 25)	Placebo (*n* = 27)	*p*-Value	Pectin (*n* = 24)	Placebo (*n* = 24)	*p*-Value
Age (years, mean ± SD)	23.4 ± 4.5	22.8 ± 4.1	0.613	69.5 ± 3.1	69.8 ± 2.4	0.723
Sex (% female)	68.0	48.1	0.148	37.5	50.0	0.383
BMI (kg/m^2^, mean ± SD)	23.2 ± 2.7	22.6 ± 2.7	0.444	25.5 ± 2.6	26.2 ± 2.8	0.334
Serum CRP (mg/L, mean ± SD)	1.7 ± 2.5	1.0 ± 1.2	0.161	1.1 ± 1.3	1.8 ± 2.1	0.203
Medication (%)	N.A.	N.A.	N.A.			
PPI				12.5	12.5	1.000
Statins				4.2	4.2	1.000
Antihypertensives				12.5	8.3	0.637
Alcohol consumption (units/week, mean ± SD)	3.5 ± 3.2	5.3 ± 5.4	0.165	8.4 ± 6.9	9.3 ± 7.1	0.667

BMI: body mass index, CRP: C-reactive protein, N.A: not applicable, and PPI: proton-pump inhibitors. Age, BMI, CRP, and alcohol consumption were compared between intervention groups with the use of an independent samples *t*-test. Sex and medication were compared between intervention groups with the use of a Pearson’s chi-square test.

**Table 2:** Relative expression of junctional complexes (e.g., tight junction related and adheren junctions) and defense- and immune-related (e.g., human defensins, cytokines, and toll-like receptor) genes in sigmoid biopsies of young adults and elderly after four weeks pectin or placebo intervention.

Cluster	Gene Name	Young Adults				Elderly			
		Pectin	Placebo	*p*-Value	Benjamini–Hochberg *p*-Value	Pectin	Placebo	*p*-Value	Benjamini–Hochberg *p*-Value
Junctional complex related genes	*TJP1 (ZO-1)*	1.15 ± 0.03	1.13 ± 0.03	0.313	0.417	1.15 ± 0.02	1.13 ± 0.02	0.195	0.260
	*OCLN*	1.19 ± 0.02	1.18 ± 0.02	0.128	0.417	1.20 ± 0.01	1.19 ± 0.02	0.184	0.260
	*CLDN2*	1.34 ± 0.07	1.36 ± 0.03	0.527	0.602	1.36 ± 0.05	1.32 ± 0.07	0.250	0.286
	*CLDN3*	1.17 ± 0.02	1.16 ± 0.02	0.245	0.417	1.18 ± 0.03	1.16 ± 0.02	0.079	0.222
	*CLDN4*	1.11 ± 0.03	1.10 ± 0.02	0.311	0.417	1.12 ± 0.02	1.10 ± 0.02	0.111	0.222
	*MLCK*	1.15 ± 0.02	1.15 ± 0.03	0.982	0.982	1.16 ± 0.02	1.14 ± 0.03	0.109	0.222
	*CDH1*	1.17 ± 0.02	1.15 ± 0.01	0.072	0.417	1.17 ± 0.03	1.17 ± 0.02	0.852	0.852
	*CTNNB1*	1.13 ± 0.01	1.12 ± 0.01	0.236	0.417	1.15 ± 0.02	1.12 ± 0.02	0.029	0.222
Defense and immune related genes	*CAMP*	1.29 ± 0.05	1.30 ± 0.05	0.630	0.770	1.32 ± 0.06	1.28 ± 0.06	0.179	0.405
	*DEFB1*	1.17 ± 0.05	1.15 ± 0.03	0.468	0.735	1.18 ± 0.05	1.16 ± 0.03	0.184	0.405
	*MUC2*	1.02 ± 0.03	1.01 ± 0.03	0.429	0.735	1.01 ± 0.03	1.01 ± 0.02	0.832	0.915
	*TFF3*	0.99 ± 0.03	0.98 ± 0.04	0.432	0.735	0.98 ± 0.05	0.98 ± 0.04	0.832	0.915
	*IL1B*	1.32 ± 0.05	1.35 ± 0.05	0.217	0.597	1.33 ± 0.04	1.31 ± 0.06	0.232	0.405
	*IL10*	1.25 ± 0.07	1.25 ± 0.03	0.856	0.856	1.27 ± 0.05	1.23 ± 0.06	0.141	0.405
	*TNF*	1.31 ± 0.05	1.35 ± 0.06	0.151	0.554	1.35 ± 0.04	1.35 ± 0.04	0.937	0.937
	*TLR1*	1.15 ± 0.05	1.18 ± 0.04	0.144	0.554	1.16 ± 0.05	1.13 ± 0.04	0.153	0.405
	*TLR2*	1.25 ± 0.06	1.26 ± 0.05	0.818	0.856	1.26 ± 0.06	1.23 ±.0.06	0.258	0.405
	*TLR4*	1.19 ± 0.03	1.21 ± 0.03	0.056	0.554	1.21 ± 0.03	1.19 ± 0.03	0.042	0.405
	*TLR6*	1.12 ± 0.06	1.29 ± 0.04	0.622	0.770	1.30 ± 0.07	1.27 ± 0.06	0.358	0.492

Glyceraldehyde-3-phosphate dehydrogenase (*GAPDH*) was used as reference gene in this table since analyses on *18S RNA* normalized gene expressions resulted in the same conclusions. Values are presented as mean ± SD. For a limited number of genes, sample sizes may differ due to technical reasons. Within age groups, genes were compared between intervention groups by independent-sample *t*-tests. *p*-values were corrected for multiple testing by calculating the false discovery rate of Benjamini–Hochberg per cluster. *TJP1* (*ZO-1*): Tight junction protein 1 (i.e., Zona Occludens-1), *OCLN*: Occludin, *CLDN*: Claudin, *MLCK*: Myosin light chain kinase, *CDH1*: Cadherin 1, *CTNNB1*: Catenin beta 1, *CAMP*: Cathelicidin antimicrobial peptide, *DEFB1*: Defensin beta 1, *MUC2*: Mucin 2, *TFF3*: Trefoil factor 3, *IL*: Interleukin, *TNF*: Tumor necrosis factor, and *TLR*: Toll-like receptor.

## Supplementary Materials

The following are available online at https://www.mdpi.com/2072-6643/11/7/1554/s1, Figure S1: Mucosal defense parameters at baseline and after four weeks of pectin (circles) or placebo (triangles) intervention in young adults and elderly. **A**: Salivary sIgA1 (g/mL) in young adults. **B**: Salivary sIgA1 (g/mL) in elderly. **C**: Salivary sIgA2 (g/mL) in young adults. **D**: Salivary sIgA2 (g/mL) in elderly. **E**: Serum IgA1 (g/mL) in young adults. **F**: Serum IgA1 (g/mL) in elderly. **G**: Serum IgA2 (g/mL) in young adults. **H**: Serum IgA2 (g/mL) in elderly. **I**: Fecal sIgA (g/L) in young adults. **J**: Fecal sIgA (g/L) in elderly. Values are presented in scatter plots with median line and IQR (25–75th interquartile range). Sample sizes vary due to drop-outs and technical reasons. Within age groups, mucosal defense parameters were compared between intervention groups with unstructured linear mixed models and correction for baseline values. IgA, Immunoglobulin A; sIgA, secretory Immunoglobulin A, Figure S2: Gastrointestinal symptoms at baseline and every week of pectin (fixed lines) and placebo (dashed lines) intervention in young adults. **A**: Abdominal pain scores. **B**: Constipation scores. **C**: Diarrhea scores. **D**: Indigestion scores. **E**: Reflux scores. Means and standard deviations are shown. Missing values at specific weeks were due to drop-outs. Gastrointestinal symptom scores were compared between intervention groups with random intercept linear mixed models and correction for baseline values. *p*-values per time point were corrected for multiple testing by calculating the false-discovery-rate (FDR) of Benjamini-Hochberg, Figure S3: Gastrointestinal symptoms at baseline and every week of pectin (fixed lines) and placebo (dashed lines) intervention in elderly. **A**: Abdominal pain scores. **B**: Constipation scores. **C**: Diarrhea scores. **D**: Indigestion scores. **E**: Reflux scores. Means and standard deviations are shown. Gastrointestinal symptom scores were compared between intervention groups with random intercept linear mixed models and correction for baseline values. *p*-values per time point were corrected for multiple testing by calculating the false-discovery-rate (FDR) of Benjamini-Hochberg, Figure S4: Representative images of tight junction protein TJP1 (green) immunofluorescence staining in sigmoid biopsy sections of a healthy young adult and healthy elderly after four weeks pectin or placebo intervention. Scale bar represents 100 μm. Blue counterstaining (DAPI) shows nuclei. TJP1: Tight junction protein 1, Figure S5: Representative images of tight junction protein occludin (red) immunofluorescence staining in sigmoid biopsy sections of a healthy young adult and healthy elderly after four weeks pectin or placebo intervention. Scale bar represents 100 μm. Blue counterstaining (DAPI) shows nuclei, Table S1: Forward and reverse primer sequences and final cDNA concentrations of all target genes, as determined in sigmoid biopsies, Table S2: Baseline characteristics of the subgroups of young adults (*n* = 22) and elderly (*n* = 22), undergoing sigmoidoscopy after the pectin or placebo intervention.

## References

[1] Bischoff S.C., Barbara G., Buurman W., Ockhuizen T., Schulzke J.-D., Serino M., Tilg H., Watson A., Wells J.M. (2014). Intestinal permeability—A new target for disease prevention and therapy. BMC Gastroenterol..

[2] König J., Wells J., Cani P.D., García-Ródenas C.L., MacDonald T., Mercenier A., Whyte J., Troost F., Brummer R.-J. (2016). Human Intestinal Barrier Function in Health and Disease. Clin. Trans. Gastroenterol..

[3] Ma T.Y., Hollander D., Dadufalza V., Krugliak P. (1992). Effect of aging and caloric restriction on intestinal permeability. Exp. Gerontol..

[4] Tran L., Meerveld B.G.-V. (2013). Age-Associated Remodeling of the Intestinal Epithelial Barrier. J. Gerontol. Ser. A Biol. Sci. Med. Sci..

[5] Thevaranjan N., Puchta A., Schulz C., Naidoo A., Szamosi J.C., Verschoor C.P., Loukov D., Schenck L.P., Jury J., Foley K.P. (2017). Age-Associated Microbial Dysbiosis Promotes Intestinal Permeability, Systemic Inflammation, and Macrophage Dysfunction. Cell Host Microbe.

[6] Siró I., Kápolna E., Kápolna B., Lugasi A. (2008). Functional food. Product development, marketing and consumer acceptance—A review. Appetite.

[7] Brouns F., Theuwissen E., Adam A., Bell M., Berger A., Mensink R.P. (2012). Cholesterol-lowering properties of different pectin types in mildly hyper-cholesterolemic men and women. Eur. J. Clin. Nutr..

[8] Tian L., Scholte J., Borewicz K., van den Bogert B., Smidt H., Scheurink A.J.W., Gruppen H., Schols H.A. (2016). Effects of pectin supplementation on the fermentation patterns of different structural carbohydrates in rats. Mol. Nutr. Food Res..

[9] Thakur B.R., Singh R.K., Handa A.K., Rao M.A. (1997). Chemistry and uses of pectin—A review. Crit. Rev. Food Sci. Nutr..

[10] Garthoff J.A., Heemskerk S., Hempenius R.A., Lina B.A.R., Krul C.A.M., Koeman J.H., Speijers G.J.A. (2010). Safety evaluation of pectin-derived acidic oligosaccharides (pAOS): Genotoxicity and sub-chronic studies. Regul. Toxicol. Pharmacol..

[11] Tian L., Bruggeman G., van den Berg M., Borewicz K., Scheurink A.J.W., Bruininx E., de Vos P., Smidt H., Schols H.A., Gruppen H. (2017). Effects of pectin on fermentation characteristics, carbohydrate utilization, and microbial community composition in the gastrointestinal tract of weaning pigs. Mol. Nutr. Food Res..

[12] Vogt L.M., Sahasrabudhe N.M., Ramasamy U., Meyer D., Pullens G., Faas M.M., Venema K., Schols H.A., de Vos P. (2016). The impact of lemon pectin characteristics on TLR activation and T84 intestinal epithelial cell barrier function. J. Funct. Foods.

[13] Sahasrabudhe N.M., Beukema M., Tian L., Troost B., Scholte J., Bruininx E., Bruggeman G., van den Berg M., Scheurink A., Schols H.A. (2018). Dietary Fiber Pectin Directly Blocks Toll-Like Receptor 2–1 and Prevents Doxorubicin-Induced Ileitis. Front. Immunol..

[14] Chen T., Kim C.Y., Kaur A., Lamothe L., Shaikh M., Keshavarzian A., Hamaker B.R. (2017). Dietary fibre-based SCFA mixtures promote both protection and repair of intestinal epithelial barrier function in a Caco-2 cell model. Food Funct..

[15] Hardy H., Harris J., Lyon E., Beal J., Foey A.D. (2013). Probiotics, Prebiotics and Immunomodulation of Gut Mucosal Defences: Homeostasis and Immunopathology. Nutrients.

[16] Rabbani G.H., Teka T., Saha S.K., Zaman B., Majid N., Khatun M., Wahed M.A., Fuchs G.J. (2004). Green banana and pectin improve small intestinal permeability and reduce fluid loss in Bangladeshi children with persistent diarrhea. Dig. Dis. Sci..

[17] Shiau S.-Y., Chang G.W. (1986). Effects of Certain Dietary Fibers on Apparent Permeability of the Rat Intestine. J. Nutr..

[18] Jiang T., Gao X., Wu C., Tian F., Lei Q., Bi J., Xie B., Wang H.Y., Chen S., Wang X. (2016). Apple-Derived Pectin Modulates Gut Microbiota, Improves Gut Barrier Function, and Attenuates Metabolic Endotoxemia in Rats with Diet-Induced Obesity. Nutrients.

[19] Ferguson L.R., Caterina R.D., Görman U., Allayee H., Kohlmeier M., Prasad C., Choi M.S., Curi R., de Luis D.A., Gil Á. (2016). Guide and Position of the International Society of Nutrigenetics/Nutrigenomics on Personalised Nutrition: Part 1—Fields of Precision Nutrition. J. Nutrigenet. Nutrigenomics.

[20] Marteau P., Seksik P. (2004). Tolerance of Probiotics and Prebiotics. J. Clin. Gastroenterol..

[21] Schwab U., Louheranta A., Törrönen A., Uusitupa M. (2006). Impact of sugar beet pectin and polydextrose on fasting and postprandial glycemia and fasting concentrations of serum total and lipoprotein lipids in middle-aged subjects with abnormal glucose metabolism. Eur. J. Clin. Nutr..

[22] François I.E.J.A., Lescroart O., Veraverbeke W.S., Marzorati M., Possemiers S., Evenepoel P., Hamer H., Houben E., Windey K., Welling G.W. (2012). Effects of a wheat bran extract containing arabinoxylan oligosaccharides on gastrointestinal health parameters in healthy adult human volunteers: A double-blind, randomised, placebo-controlled, cross-over trial. Br. J. Nutr..

[23] Holscher H.D., Caporaso J.G., Hooda S., Brulc J.M., Fahey G.C., Swanson K.S. (2015). Fiber supplementation influences phylogenetic structure and functional capacity of the human intestinal microbiome: follow-up of a randomized controlled trial. Am. J. Clin. Nutr..

[24] Van Wijck K., van Eijk H.M.H., Buurman W.A., Dejong C.H.C., Lenaerts K. (2011). Novel analytical approach to a multi-sugar whole gut permeability assay. J. Chromatogr. B Anal. Technol. Biomed. Life Sci..

[25] Van Wijck K., Verlinden T.J.M., van Eijk H.M.H., Dekker J., Buurman W.A., Dejong C.H.C., Lenaerts K. (2013). Novel multi-sugar assay for site-specific gastrointestinal permeability analysis: A randomized controlled crossover trial. Clin. Nutr..

[26] Svedlund J., Sjödin I., Dotevall G. (1988). GSRS--a clinical rating scale for gastrointestinal symptoms in patients with irritable bowel syndrome and peptic ulcer disease. Dig. Dis. Sci..

[27] Rinsma N.F., Farré R., Troost F.J., Elizalde M., Keszthelyi D., Helyes Z., Masclee A.A., Conchillo J.M. (2017). Exploration of the Esophageal Mucosal Barrier in Non-Erosive Reflux Disease. Int. J. Mol. Sci..

[28] Pijls K.E., Jonkers D.M.A.E., Elizalde M., Drittij-Reijnders M.-J., Haenen G.R., Bast A., Masclee A.A.M., Koek G.H. (2016). Is intestinal oxidative stress involved in patients with compensated liver cirrhosis?. Ann. Hepatol..

[29] Elamin E., Masclee A., Troost F., Pieters H.-J., Keszthelyi D., Aleksa K., Dekker J., Jonkers D. (2014). Ethanol Impairs Intestinal Barrier Function in Humans through Mitogen Activated Protein Kinase Signaling: A Combined In Vivo and In Vitro Approach. PLoS ONE.

[30] Russo F., Linsalata M., Clemente C., Chiloiro M., Orlando A., Marconi E., Chimienti G., Riezzo G. (2012). Inulin-enriched pasta improves intestinal permeability and modifies the circulating levels of zonulin and glucagon-like peptide 2 in healthy young volunteers. Nutr. Res..

[31] Salden B.N., Troost F.J., Wilms E., Truchado P., Vilchez-Vargas R., Pieper D.H., Jáuregui R., Marzorati M., van de Wiele T., Possemiers S. (2018). Reinforcement of intestinal epithelial barrier by arabinoxylans in overweight and obese subjects: A randomized controlled trial: Arabinoxylans in gut barrier. Clin. Nutr..

[32] Krumbeck J.A., Rasmussen H.E., Hutkins R.W., Clarke J., Shawron K., Keshavarzian A., Walter J. (2018). Probiotic Bifidobacterium strains and galactooligosaccharides improve intestinal barrier function in obese adults but show no synergism when used together as synbiotics. Microbiome.

[33] Ganda Mall J.P., Löfvendahl L., Lindqvist C.M., Brummer R.J., Keita Å.V., Schoultz I. (2018). Differential effects of dietary fibres on colonic barrier function in elderly individuals with gastrointestinal symptoms. Sci. Rep..

[34] Sun Y., He Y., Wang F., Zhang H., de Vos P., Sun J. (2017). Low-methoxyl lemon pectin attenuates inflammatory responses and improves intestinal barrier integrity in caerulein-induced experimental acute pancreatitis. Mol. Nutr. Food Res..

[35] Woof J.M., Kerr M.A. (2006). The function of immunoglobulin A in immunity. J. Pathol..

[36] Lecerf J.-M., Dépeint F., Clerc E., Dugenet Y., Niamba C.N., Rhazi L., Cayzeele A., Abdelnour G., Jaruga A., Younes H. (2012). Xylo-oligosaccharide (XOS) in combination with inulin modulates both the intestinal environment and immune status in healthy subjects, while XOS alone only shows prebiotic properties. Br. J. Nutr..

[37] Walton G.E., Lu C., Trogh I., Arnaut F., Gibson G.R. (2012). A randomised, double-blind, placebo controlled cross-over study to determine the gastrointestinal effects of consumption of arabinoxylan-oligosaccharides enriched bread in healthy volunteers. Nutr. J..

[38] Vulevic J., Juric A., Tzortzis G., Gibson G.R. (2013). A Mixture of trans-Galactooligosaccharides Reduces Markers of Metabolic Syndrome and Modulates the Fecal Microbiota and Immune Function of Overweight Adults. J. Nutr..

[39] Kato T., Fukuda S., Fujiwara A., Suda W., Hattori M., Kikuchi J., Ohno H. (2014). Multiple Omics Uncovers Host–Gut Microbial Mutualism During Prebiotic Fructooligosaccharide Supplementation. DNA Res..

[40] Lim B.O., Yamada K., Nonaka M., Kuramoto Y., Hung P., Sugano M. (1997). Dietary Fibers Modulate Indices of Intestinal Immune Function in Rats. J. Nutr..

[41] Holscher H.D., Doligale J.L., Bauer L.L., Gourineni V., Pelkman C.L., Fahey G.C., Swanson K.S. (2014). Gastrointestinal tolerance and utilization of agave inulin by healthy adults. Food Funct..

[42] Bergamasco C., Horie L.M., Torrinhas R.S., Waitzberg D.L. (2015). High-Fiber Orange Juice as a Nutrition Supplement in Women. J. Perenter. Enteral. Nutr..

