# Supplementary material for: The Impact of Pectin Supplementation on Intestinal Barrier Function in Healthy Young Adults and Healthy Elderly
Source: Nutrients. 2019 Jul 9;11(7):1554. doi: 10.3390/nu11071554 (PMC6683049; doi:10.3390/nu11071554)
Supplement: Supplementary file 1 [file nutrients-11-01554-s001.pdf]

Supplementary Materials

Table S1. Forward and reverse primer sequences and final cDNA concentrations of all target genes, as determined in sigmoid biopsies.

Name	Gene description	Primer sequences	Final cDNA concentration
CAMP	Cathelicidin antimicrobial peptide	5′-AGGATTGTGACTTCAAGAAGGACG-3′<br>5′-GTTTATTTCTCAGAGCCCAGAAGC-3′	80 ng/µl
CDH1	Cadherin 1	5′-CACCTGGAGAGAGGCCGCGT -3′<br>5′-AACGGAGGCCTGATGGGGCG -3′	20 ng/µl
CLDN2	Claudin 2	5′-AACTACTACGATGCCTACC-3′<br>5′-GAACTCACTCTTGACTTTGG -3′	20 ng/µl
CLDN3	Claudin 3	5′-TTCATCGGCAGCAACATCATC-3′<br>5′-CGCCTGAAGGTCCTGTGG-3′	20 ng/µl
CLDN4	Claudin 4	5′-ACAGACAAGCCTTACTCC-3′<br>5′-GGAAGAACAAAGCAGAG-3′	20 ng/µl
CTNNB1	Catenin beta 1	5′-GTGCTATCTGTCTGCTCTAGTA -3′<br>5′-CTTCCTGTTTAGTTGCAGCATC -3′	20 ng/µl
DEFB1	Defensin beta 1	5′-CTCTGTCAGCTCAGCCTC-3′<br>5′-CTTGCAGCACTTGGCCTTCCC-3′	20 ng/µl
GAPDH	Glyceraldehyde-3-phosphate dehydrogenase	5′-TGCACCACCAACTGCTTAGC-3′<br>5′-GGCATGGACTGTGGTCATGAG-3′	20 ng/µl
IL1B	Interleukin 1 beta	5′-AAACAGATGAAGTGCTCCTTCCAGG-3′<br>5′-TGGAGAACACCACTTGTTGCTCCA-3′	40 ng/µl
IL10	Interleukin 10	5′-TCAGGGTGGCGACTCTAT-3′<br>5′-TGGGCTTCTTTCTAAATCGTTC-3′	80 ng/µl
MUC2	Mucin 2	5′-GTCAACCCTGCCGACACCTG-3′<br>5′-ACTCACACCAGTAGAAAGGACAGC-3′	20 ng/µl
MLCK	Myosin light chain kinase	5-GCCTGACCACGAATATAAGTT-3′<br>5′-GCTCCTTCTCATCATCATCTG-3′	20 ng/µl
OCLN	Occludin	5′-TCAGGGAATATCCACCTATCACTTCAG-3′<br>5′-CATCAGCAGCAGCCATGTACTCTTCAC-3′	20 ng/µl
TFF3	Trefoil factor 3	5′-CTTGCTGTCCTCCAGCTCT-3′<br>5′-CCGGTTGTTGCACTCCTT-3′	20 ng/µl
TJP1 (ZO-1)	Tight junction protein 1	5′-AGGGGCAGTGGTGGTTTTCTGTTCTTTC-3′<br>5′-GCAGAGGTCAAAGTTCAAGGCTCAAGAGG-3′	20 ng/µl
TLR1	Toll like receptor 1	5′-CAGTGTCTGGTACACGCATGGT-3′<br>5′-TTTCAAAAACCGTGTCTGTTAAGAGA-3′	80 ng/µl
TLR2	Toll like receptor 2	5′-GCCAAAGTCTTGATTGATTGG-3′	20 ng/µl

		5′-TATACCACAGGCCATGGAAAC-3′	
TLR4	Toll like receptor 4	5′-CCTGCGTGAGACCAGAAAGC-3′	80 ng/µl
		5′-TCAGCTCCATGCATTGATAAGTAATA-3′	
TLR6	Toll like receptor 6	5′-GAAGAAGAACAACCCTTTAGGATAGC-3′	20 ng/µl
		5′-AGGCAAACAAAATGGAAGCTT-3′	
TNF	Tumor Necrosis Factor	5′-CCGAGTGACAAGCCTGTAGC-3′	40 ng/µl
		5′-GAGGACCTGGGAGTAGATGAG-3′	
18S RNA	18S ribosomal RNA	5′-GTAACCCGTTGAACCCCATT-3′	20 ng/µl
		5′-CCATCCAATCGGTAGTAGCG-3′	

Table S2. Baseline characteristics of the subgroups of young adults (n=22) and elderly (n=22), undergoing sigmoidoscopy after the pectin or placebo intervention

	**Young adults (n=22)**			**Elderly (n=22)**		
	**Pectin (n=12)**	**Placebo (n=10)**	***P*-value**	**Pectin (n=12)**	**Placebo (n=10)**	***P*-value**
Age (yrs, mean ± SD)	23.9 ± 4.6	24.2 ± 5.4	0.893	70.4 ± 3.0	70.7 ± 2.8	0.784
Sex (% female)	58.3	30.0	0.184	41.7	20.0	0.277
BMI (kg/m², mean ± SD)	22.3 ± 2.8	23.7 ± 3.2	0.299	25.6 ± 2.6	27.3 ± 1.7	0.083
Serum CRP (mg/L, mean ± SD)	1.8 ± 1.8	0.6 ± 0.7	0.061	0.7 ± 0.6	1.8 ± 1.6	0.032
Medication (%)						
PPI				8.3	20.0	0.427
Statins	N.A.	N.A.	N.A.	8.3	0	0.350
Antihypertensives				0	10	0.262
Alcohol consumption (units/week, mean ± SD)	3.0 ± 2.7	6.3 ± 6.3	0.156	5.5 ± 5.0	10.8 ± 6.8	0.049

BMI: body mass index, CRP: C-reactive protein, N.A: not applicable, PPI: proton-pump inhibitors. Age, BMI, CRP and alcohol consumption were compared between intervention groups with the use of an independent samples t-test. Sex and medication were compared between intervention groups with the use of a Pearson Chi-square test.

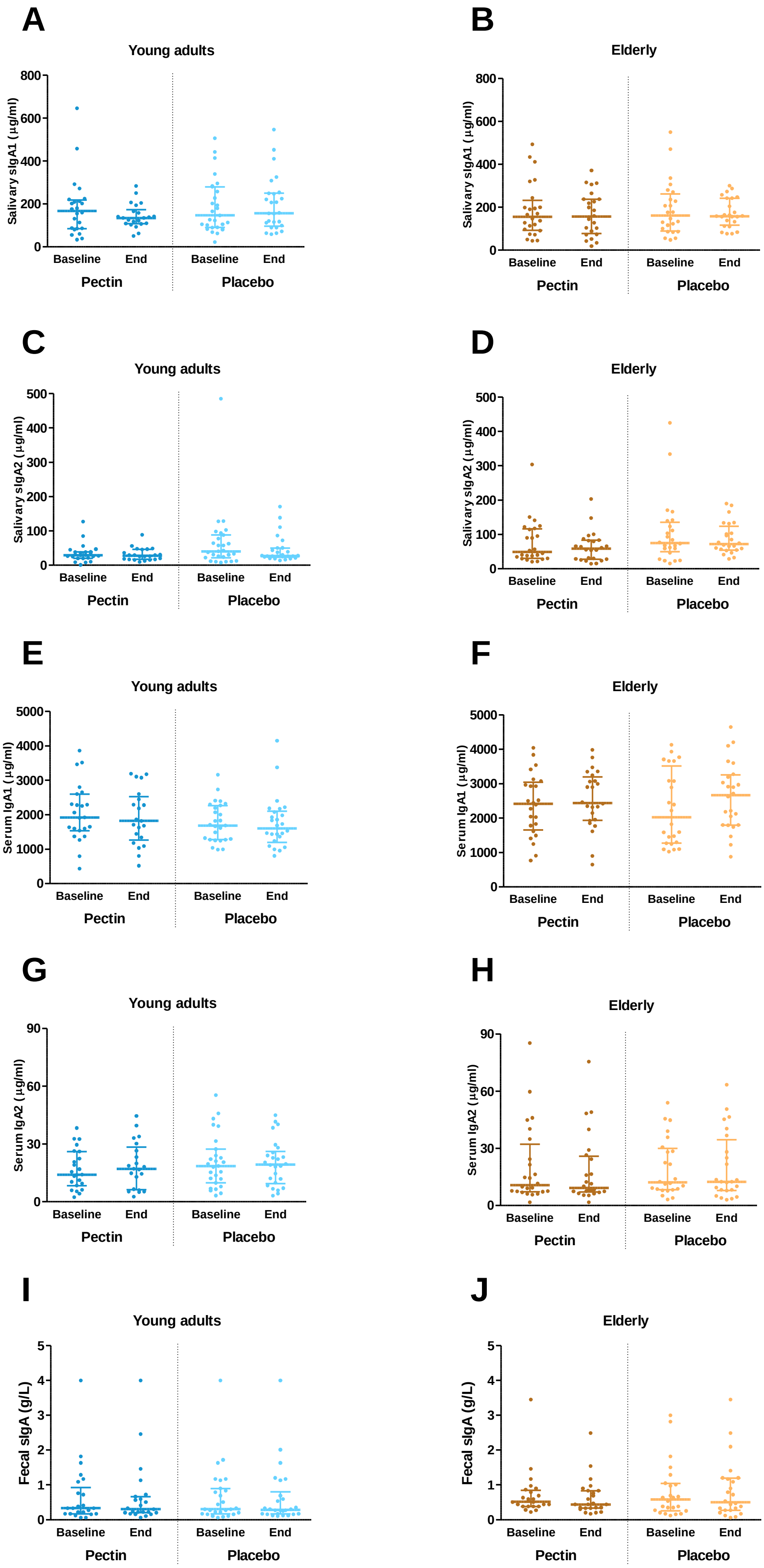
A
Young adults
Salivary sIgA1 (µg/ml)
0
200
400
600
800
Baseline
End
Baseline
End
Pectin
Placebo
B
Elderly
Salivary sIgA1 (µg/ml)
C
Young adults
Salivary sIgA2 (µg/ml)
0
100
200
300
400
500
D
Elderly
Salivary sIgA2 (µg/ml)
E
Young adults
Serum IgA1 (µg/ml)
0
1000
2000
3000
4000
5000
F
Elderly
Serum IgA1 (µg/ml)
G
Young adults
Serum IgA2 (µg/ml)
0
30
60
90
H
Elderly
Serum IgA2 (µg/ml)
I
Young adults
Fecal sIgA (g/L)
0
1
2
3
4
5
J
Elderly
Fecal sIgA (g/L)

Figure S1. Mucosal defense parameters at baseline and after four weeks of pectin (circles) or placebo (triangles) intervention in young adults and elderly. A: Salivary sIgA1 (g/ml) in young adults. B: Salivary sIgA1 (g/ml) in elderly. C: Salivary sIgA2 (g/ml) in young adults. D: Salivary sIgA2 (g/ml) in elderly. E: Serum IgA1 (g/ml) in young adults. F: Serum IgA1 (g/ml) in elderly. G: Serum IgA2 (g/ml) in young adults. H: Serum IgA2 (g/ml) in elderly. I: Fecal sIgA (g/L) in young adults. J: Fecal sIgA (g/L) in elderly. Values are presented in scatter plots with median line and IQR (25-75th interquartile range). Sample sizes vary due to drop-outs and technical reasons. Within age groups, mucosal defense parameters were compared between intervention groups with unstructured linear mixed models and correction for baseline values. IgA, Immunoglobulin A; sIgA, secretory Immunoglobulin A.

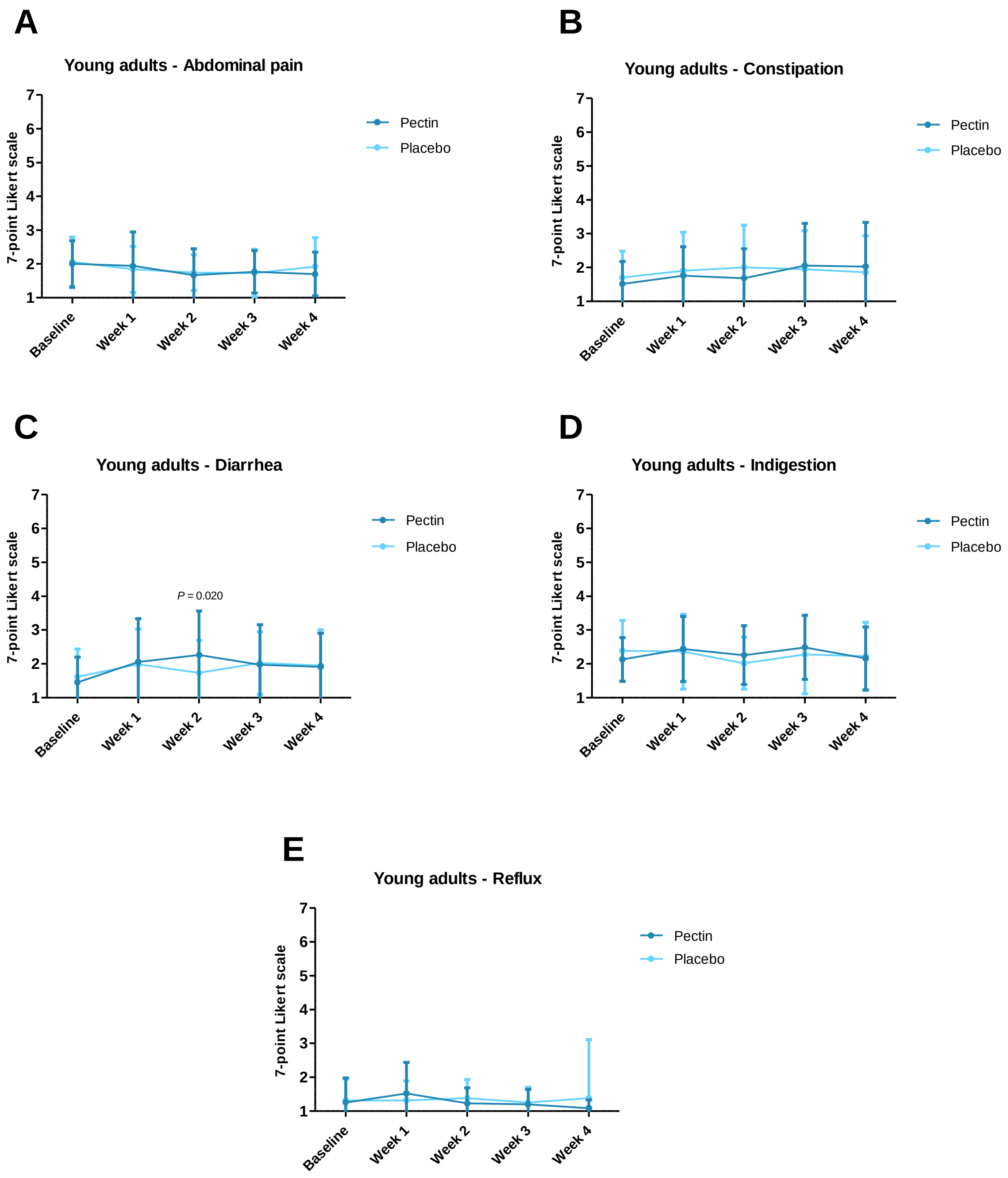

Figure S2. Gastrointestinal symptoms at baseline and every week of pectin (fixed lines) and placebo (dashed lines) intervention in young adults. A: Abdominal pain scores. B: Constipation scores. C:

Diarrhea scores. D: Indigestion scores. E: Reflux scores. Means and standard deviations are shown. Missing values at specific weeks were due to drop-outs. Gastrointestinal symptom scores were compared between intervention groups with random intercept linear mixed models and correction for baseline values. *P*-values per time point were corrected for multiple testing by calculating the false-discovery-rate (FDR) of Benjamini-Hochberg.

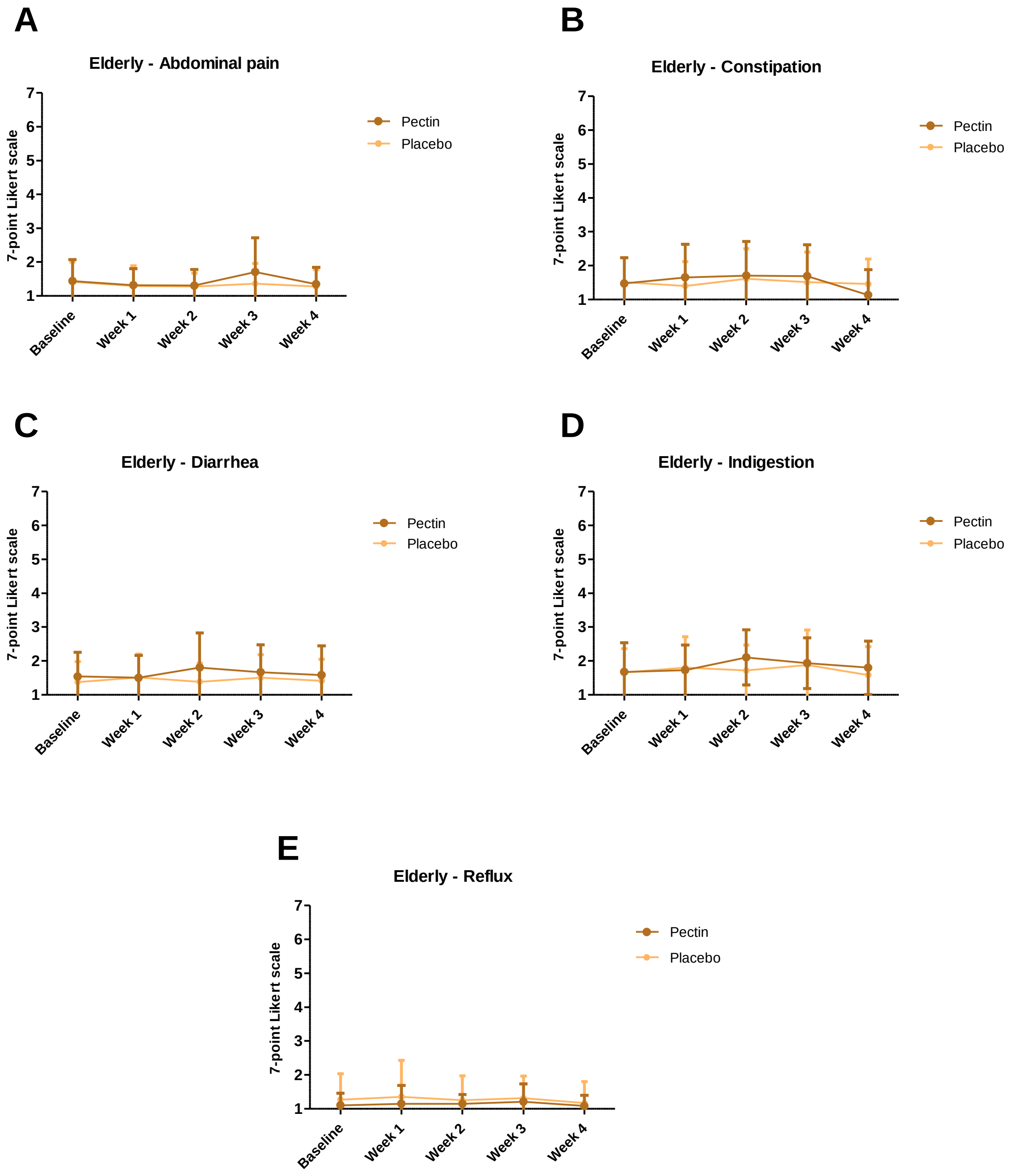

Figure S3. Gastrointestinal symptoms at baseline and every week of pectin (fixed lines) and placebo (dashed lines) intervention in elderly. A: Abdominal pain scores. B: Constipation scores. C: Diarrhea scores. D: Indigestion scores. E: Reflux scores. Means and standard deviations are shown. Gastrointestinal symptom scores were compared between intervention groups with random intercept linear mixed models and correction for baseline values. *P*-values per time point were corrected for multiple testing by calculating the false-discovery-rate (FDR) of Benjamini-Hochberg.

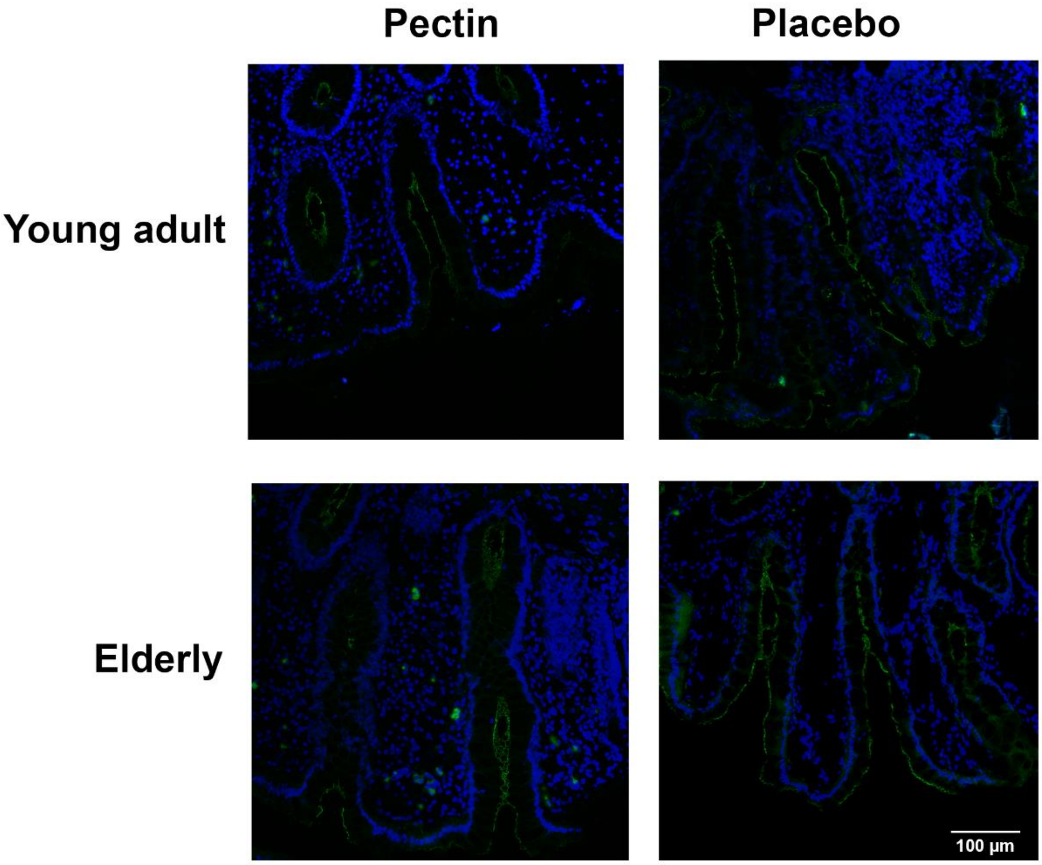

Figure S4. Representative images of tight junction protein TJP1 (green) immunofluorescence staining in sigmoid biopsy sections of a healthy young adult and healthy elderly after four weeks pectin or placebo intervention. Scale bar represents 100 µm. Blue counterstaining (DAPI) shows nuclei. TJP1: Tight junction protein 1.

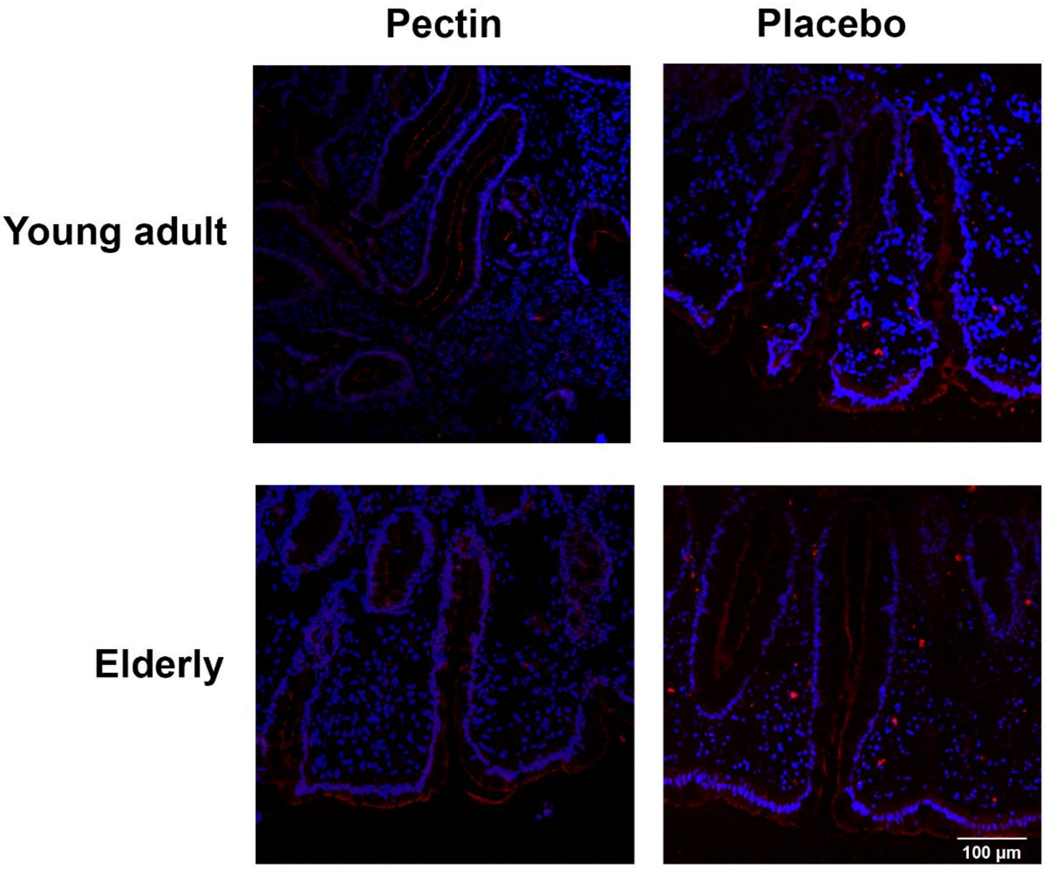

Figure S5. Representative images of tight junction protein occludin (red) immunofluorescence staining in sigmoid biopsy sections of a healthy young adult and healthy elderly after four weeks pectin or placebo intervention. Scale bar represents 100 µm. Blue counterstaining (DAPI) shows nuclei.
